# Court-Mandated Patients’ Perspectives on the Psychotherapist’s Dual Loyalty Conflict – Between Ally and Enemy

**DOI:** 10.3389/fpsyg.2020.592638

**Published:** 2021-01-06

**Authors:** Helene Merkt, Tenzin Wangmo, Félix Pageau, Michael Liebrenz, Corinne Devaud Cornaz, Bernice Elger

**Affiliations:** ^1^Insitute for Biomedical Ethics, University of Basel, Basel, Switzerland; ^2^Department of Forensic Psychiatry, Institute of Forensic Medicine, University of Bern, Bern, Switzerland; ^3^Unité Thérapeutique, Centre de Psychiatrie Forensique, Réseau Fribourgeois de Santé Mentale, Fribourg, Switzerland

**Keywords:** dual role, dual loyalty, triangular relationship, prison, offender, therapeutic alliance, coercion, limited confidentiality

## Abstract

**Background:**

Mental health professionals working in correctional contexts engage a double role to care and control. This dual loyalty conflict has repeatedly been criticized to impede the development of a high-quality alliance. As therapeutic alliance is a robust predictor of outcome measures of psychotherapy, it is essential to investigate the effects of this ethical dilemma.

**Methods:**

This qualitative interview study investigates patients’ perceptions of their therapists’ dual role conflict in court-mandated treatment settings. We interviewed 41 older incarcerated persons using a semi-structured interview guide, the interviews were subsequently analyzed following thematic analysis.

**Results:**

We first present the patients’ perceptions of their treating psychotherapist’s dual loyalty conflict, which was linked to their overall treatment experience. In a second step, we outline the study participants’ reasons for this judgment, which were most commonly linked to feelings of trust or betrayal. More specifically, they named certain therapist characteristics and activities that enabled them to develop a trustful therapeutic alliance, which we grouped into four topics: (1) respecting the patient’s pace and perceived coercion; (2) patient health needs to be first priority; (3) clarity in roles and responsibilities; and (4) the art of communication – between transparency and unchecked information sharing.

**Discussion:**

Developing a high quality alliance in mandatory offender treatment is central due to its relationship with recovery and desistance. Our findings show that some therapists’ characteristics and activities attenuate the negative impact of their double role on the development and maintenance of the alliance. To increase the effectiveness of court-mandated treatments, we need to support clinicians in dealing with their dual role to allow the formation of a high quality therapeutic alliance. Our qualitative interview study contributed to this much-needed empirical research on therapist’ characteristics promoting a trustful relationship in correctional settings.

## Introduction

The goal of court-mandated treatment orders is to reduce the risk of recidivism in mentally ill persons in detention (Dowling et al., 2018). It is therefore crucial to enhance effectiveness of these interventions to protect the society. However, psychological practice in correctional settings is criticized for not meeting the standards of evidence-based practice, amongst others, due to the dual loyalty conflict (Gannon and Ward, 2014; Goulet et al., 2019). The therapists’ dual role when treating an incarcerated person, to provide care and at the same time to control, challenges the development of a high-quality therapeutic alliance with the patient (Cervantes and Hanson, 2013; Wittouck and Vander Beken, 2019). As therapeutic alliance is a robust predictor of outcome measures of psychotherapy (Martin et al., 2000; Horvath et al., 2011; Fluckiger et al., 2015, 2018), it is essential to investigate the influence of the therapist’s dual role on the alliance to reach the goal of court-mandated treatment orders.

The therapeutic alliance is one of the common factors of psychotherapeutic practice overarching different techniques (Mulder et al., 2017; Horvath, 2018). It can therefore be assumed that this therapeutic element is likewise important in therapy with persons who are incarcerated (Blasko et al., 2018). The greatest differences in the quality of the alliance have been attributed to therapists’ contributions (Ackerman and Hilsenroth, 2003; Baldwin et al., 2007; Del Re et al., 2012). For instance, therapists’ abilities to display genuineness and empathy are strong moderators of the alliance-outcome relationship (Nienhuis et al., 2018). However, it is unclear how specific circumstances and institutions influence the processes of developing and managing such an alliance (Fluckiger et al., 2015; Horvath, 2018). Correctional settings come with specific challenges and characteristics to treatment (Meyer et al., 2019) such as handling limited confidentiality during interactions with representatives of the justice system (e.g., therapists have to provide a report on a person’s treatment progress and the risk of further offending) or being asked to manage risk and restrictions posed on this population (e.g., privileges are granted and revoked by legal authorities, however, these decisions may be based on therapists assessment on the therapy progress) (Dowling et al., 2018). This requires additional skills specific to this environment to create a therapeutic alliance with their clients.

Mental health professionals face loyalty conflicts when working in a correctional context (Magaletta et al., 2007; Cervantes and Hanson, 2013). As representatives of the mental health care system working with patients within the justice system, they need to balance individual patient’s well-being against others’ safety (Goulet et al., 2019). Based on international standards, mental health care in prisons should be under the authority of the ministry/department of health instead of under the ministry/department of justice. This for reasons of improving quality of health care in prisons but also to enhance public health in general (see Coyle, 2014). In reality, this standard is often not implemented and many services are still affiliated with the justice department with the consequence that it is often under shared responsibility of both the health and the justice system (Salize and Dressing, 2008). Pont et al. (2012) further argue that health care professionals who are directly employed by the justice system face a stronger dual loyalty conflict. Switzerland is a particularly interesting country in this sense, as the French speaking region mainly employs their mental health care professionals through the public health care system while some German speaking region employs these professionals more frequently via the justice system. The strength of the subjectively experienced dual loyalty conflict and pressure put on health personnel by the justice system might therefore differ between the two language regions. Empirical evidence supporting this hypothesis is lacking.

Nevertheless, the tension arising from these differing goals, care versus control, reveals itself in the therapeutic alliance, most dominantly regarding coercion and medical confidentiality (Wittouck and Vander Beken, 2019). Some authors have argued that it is crucial how the client perceives coercion and the exercise of power by the therapist. Studies have shown that perceived coercion is negatively correlated with patient ratings of therapeutic alliance (Manchak et al., 2014; Sheehan and Burns, 2011). At the same time, others noted that legal coercion cannot be equated with perceived coercion as there is different sources of coercion (Hachtel et al., 2019; Urbanoski, 2010). Social pressures can also arise through informal (family and friends) or formal (e.g. employer) influences. Further, the relation between these “objective” measures and perceived coercion to enter and participate in treatment is unclear (Prendergast et al., 2009; Wild, 2006). Suggesting that other factors are intermediary to the way coercion is perceived on patient’s side. In the same line, Höfer et al. (2015) revealed that alliance ratings were independent of the patient’s legal status (i.e., general psychiatry wards and forensic units). Hachtel et al. (2019) provide an explanation for this phenomenon and argue that the quality of the relationship might be closely linked to the level of perceived coercion. However, it is unclear which and how specific therapist activities influence coercion and alleviate the impact of the dual loyalty conflict on the therapeutic alliance.

Previous research in correctional psychology highlights the importance of transparency regarding the therapist’s role in risk management (Dowling et al., 2018; Merkt et al., unpublished), perceived coercion (Hotzy and Jaeger, 2016) as well as in relation to limits to confidentiality (Gannon and Ward, 2014; Elger et al., 2015a, b). Others have emphasized that a collaborative but directive style was beneficial (Ross et al., 2008; Blasko et al., 2018; Jeglic and Katsman, 2018; Meyer et al., 2019) while a harsh confrontational and authoritarian style was negatively linked to alliance measures (Marshall and Serran, 2004; Meyer et al., 2019). Wittouck and Vander Beken (2019) proposed that these preliminary findings could be subsumed under the procedural justice theory. According to their approach, therapists who follow the six principles of fairness, voice, validation, respect, motivation (or trust), and information are able to reconcile care and control. However, empirical evidence on the therapeutic alliance in offender therapy that could support this approach is still scant (Skeem et al., 2007; Ross et al., 2008; Polaschek and Ross, 2010; Blasko et al., 2018).

Lastly, the older population is drastically rising within correctional settings (Luallen and Cutler, 2017). They comprise a population of high somatic and mental health needs and therefore considerably impact health care services (Fazel et al., 2006; Fazel and Baillargeon, 2011; Fazel and Seewald, 2012; Di Lorito et al., 2018). In the Swiss context, the rising number of older persons in forensic settings is mainly fed by persons mandated to treatment. That is, the number of older persons sentenced to mandated treatment [e.g., under Art. 59 Swiss Criminal Code (SCC)] comprised 8.7% in 1999 and rose to 17.8% in 2019 (Bundesamt für Statistik, 2020a). In contrast, within the same time period, the number of persons over the age of 49 sentenced to a penal sentence rose only from 8 to 9.5% (Bundesamt für Statistik, 2020b). They are therefore a population that requires intensive resources from the forensic mental health services but the data to guide treatment planning for this specific group is scarce (Williams et al., 2012). Some authors have highlighted that there are particular challenges in the psychotherapeutic practice with this aging population such as the changing perspectives toward past crimes due to the little life time remaining or fear of dying in prison (Avieli, 2020). It is therefore important to shed light into the treatment experiences and needs of older persons who are legally referred to involuntary treatment. Our qualitative interview study fills an important gap by investigating older patients’ perceptions of their therapists’ dual role conflict in court-mandated treatment settings and thereby contributes to much-needed alliance research in therapy with incarcerated persons.

## Materials and Methods

This qualitative article follows the “Journal article reporting guidelines” for qualitative research by Levitt et al. (2018), which incorporates qualitative studies reporting guidelines such as COREQ-32 (Tong et al., 2007).

### Study Design

This qualitative study is part of a larger Swiss-wide research project on aging experiences and mental health of older persons living in detention (“Agequake in Prisons 2”). As part of the larger project, we not only gathered qualitative data from older persons in prison (described below) but also professional stakeholders, and quantitative information on older persons’ mental health condition from medical records and standardized surveys. As older persons in prison are a minority and there is relatively little data on the mental health of this population (Moschetti et al., 2015), the overall goal of the qualitative data collection was to gain insights into their experiences on aging in prison, living with a mental disorders, and their perspectives on prison mental health care. As these are complex social processes that we, to date, know little about, we applied an explorative qualitative approach to capture these social phenomena. Further, as this larger research project covered multiple issues addressing several specific research questions, only a portion of the results relevant for this paper are presented here. Namely, the participants’ perceptions of their therapists’ dual role conflict. [Please see our other publications for more findings from our research project (e.g., Haesen et al., in press; Merkt et al., unpublished).]

TW and BE conceptualized the research project. Both have many years of research experience on the topic of older persons living in detention as well as in employing qualitative methodology (Elger et al., 2015a, b; Wangmo et al., 2015, 2016a,b). Two research assistants completing their doctoral education conducted the interviews, out of which one was HM. They were trained in qualitative data collection and received supervision throughout the data collection process. Ethics approval was obtained from the regional ethics committee (Ethikkommission Nordwest- und Zentralschweiz) which was followed by other local ethics committees. On the topic of dual loyalty, a manuscript delineating the perspective of stakeholders has been written (Merkt et al., unpublished).

### Data Collection

Face-to-face interviews were conducted between December 2017 and December 2018 with persons receiving mental health care in Swiss correctional institutions. The inclusion criteria were (1) person sentenced to prison confinement, (2) age 50 years and older, and (3) at least one contact with mental health services. Exclusion criteria were (1) mental state too instable and (2) prison administration does not allow the person to participate (e.g., due to dangerousness or solitary confinement). The age cut-off 50 was applied for reasons of accelerated aging, that is, persons living in detention tend to depict poorer health status at a younger age when compared to persons of similar age group in the community (Fazel et al., 2001; Loeb et al., 2008; Hayes et al., 2012; Combalbert et al., 2016; Di Lorito et al., 2018; Greene et al., 2018; Merkt et al., 2020).

We included participants from institutions that housed adults sentenced to long-term imprisonment (please see section “Context Information” for information on the Swiss legal context and type of settings). We excluded correctional institutions that housed juvenile or remand prisoners exclusively as well as administrative detention centers (centers housing migrants for deportation). Further, psychiatric, therapeutic, and penal institutions from the two major language regions (French and German speaking) were included, the Italian speaking language region was excluded.

All participants were contacted either through the prison administration or the mental health service. We do not know the refusal rates, as participants were recruited through our contact persons in the participating correctional institutions and the internal recruiting processes differed. Study information and informed consent was previously handed out to the participants by our contact person in those settings. At the scheduled time and place of the interview, the researchers explained the purpose of the study, clarified that all data was treated confidentially, and that refusal was possible at all times. Thereafter, written informed consent was obtained. There was no compensation provided for study participation. When the time of the interview interfered with the participant’s work time, the correctional institution organized monetary substitution for the lost work hours.

The interviews with the study participants were semi-structured and followed an interview guide specifically developed for the purpose of this study. The open-ended questions within this interview guide covered topics on (a) personal circumstances and social networks, (b) experience of aging in the prison context [e.g., relationship with younger persons in detention, satisfaction with work and free time activities offered, perception of prison environment, future plans (during and after imprisonment)], (c) access to and quality of mental health care (e.g., types of interventions, frequency, and duration of treatments), (d) satisfaction with mental health care (specific aspects of the intervention that helped/impeded therapy progress), (e) mental well-being (e.g., perception of their current mental well-being, questions on possible stigma due to mental health issues), and (f) experiences with risk assessments.

Thoughts on the dual loyalty conflict were encouraged through the use of an elicitation technique. Elicitation techniques are visualization tasks that are particularly useful to inquire contents and topics that are difficult to inquire with direct explicit interview questions such as abstract concepts or controversial topics. They are used to facilitate the conversation on the topic of interest, to provoke the expression of ideas, views, or values (Copeland and Agosto, 2012; Barton, 2015). We asked participants to position their mental health professional using a coin within a triangle that represented the dual loyalty conflict. More precisely, at a certain stage during the interview, we presented the paper with the triangle graphic (see Figures 2–4 for examples) and passed a coin to the participant, asking them to position the MHP within it. We used this positioning task as a starting point to facilitate the conversation on their experiences with their MHP’s dual role. Thus, we did not explain our understanding of the triangle to the participant but used it to inquire the participants’ understanding of the MHP’s dual role. Out of the 41 participants, two participants did not complete the elicitation technique for personal reasons.

Interviewer and participant met the first time on the day of the interview, thus, there was no relationship prior to data collection. Only one interview meeting took place with each participant and no repeat interview was done. All interviews were audio-recorded upon the written informed consent of the participant. Field notes were taken after each interview. Interviews were held in the language spoken by the participant, either French, English, German, or Swiss German. Thereafter the interviews were transcribed verbatim in the language of the interview, except for Swiss German interviews, which were transcribed in Standard German. Swiss German is a spoken dialect and it is common practice to use Standard German in writing. The interviews were checked for the quality and accuracy of the transcriptions, during which identifying information were anonymized. Interview transcripts were not returned to the participants for checking.

In total, we conducted 57 interviews, of which seven were excluded mostly due to poor data quality. We based our decision to stop data collection on the principle of data saturation. We identified data saturation when the ability to obtain no additional new information has been attained, further new code is no longer feasible, and there is enough information to replicate the study (Fusch and Ness, 2015). To be able to identify when data saturation was reached for each linguistic region, we conducted data analysis along the on-going data collection and were therefore able to include more participants if needed.

From the total usable data corpus of 50 interviews, 9 interviewees were excluded for the data analysis for this specific manuscript because, they were receiving mental health care but were sentenced to a penal sentence (please see further explanation of differences between “measures” and “penalties” in the section “Context Information” below). Some regulations in regards to mental health care apply for persons sentenced under measures and penalties. For instance, during the treatment of a person sentenced to a penalty, the regular medical confidentiality applies while confidentiality is limited in the treatment of a person sentenced to a measure. See Table 1 for more details on participants’ characteristics.

**TABLE 1:** Participant characteristics (*N* = 41).

**Institutions**		
Forensic-Psychiatric Institutions	14	Participants
Penal Institutions	27	
**Gender**		
Female	2	Participants
Male	39	
**Language region**		
French	18	Participants
German	23	
**Age**		
Average	62	Years
Range	50–76	
Standard deviation	6.92	
**Interview length**		
Average	69	Minutes
Range	16–120	
Standard deviation	25.55	

### Context Information

The SCC regulates penal law on a national level, sanctions that are imposed for certain crimes are therefore similar across the nation. However, the imposition of sentences is regulated on a federal level. Each state (canton) orchestrates the precise execution of the sentences. Thus, some aspects will vary on a cantonal level such as the settings and placement of mentally ill persons (Fink, 2018). This being said, we will first depict some important differences in the SCC, which are important in light of our analysis. In a second step, we will briefly outline the characteristics of the settings, in which incarcerated persons are housed, which has implications for the mental health care received.

The SCC distinguishes between penalties (Strafen) and measures (Massnahmen). Measures can be imposed when penalty alone is not sufficient to counter the risk of further offending and the offender requires treatment or treatment is required in the interest of public safety (SCC). To impose a measure, the court bases its decision on an expert assessment which comprises estimations of (a) the necessity and the prospects of success of any treatment of the offender; (b) the nature and the probability of possible additional offenses; and (c) the ways in which the measure may be implemented. Measures are reassessed at regular time intervals and release is granted based on the fulfillment of the requirements of the parole boards and the risk for further felonies. For all measures, criminal responsibility can be diminished, however, it is not a *sine qua non* condition for the judge to impose a therapeutic measure.

In our sample, we included persons sentenced to measures under Art. 59 (in-patient therapeutic measures), Art. 63 (out-patient treatment), and Art. 64 (indefinite incarceration). The basic conditions outlined in the previous paragraph concern all measures while certain aspects are specific to each type. For instance, Art. 59 and 63 can be ordered if the person suffers from a severe mental disorder that stands in direct connection with the crime committed and it is expected that the measure will reduce the risk to reoffend. Art. 59 requires the person to be incarcerated while a person sentenced under Art. 63 receives ambulatory mandatory treatment. They can either live in the community or be placed in a correctional institution due to an additional penalty. We included only persons that were incarcerated at the time of data collection.

Article 64 can be imposed on a person who committed a crime comprising another person’s integrity (e.g., sexual offenses and murder). The person suffers from a permanent or long-term mental disorder of considerable gravity that was a factor in the offense and it is seriously expected that the offender will reoffend. In such cases, ordering of a measure in accordance with Art. 59 does not promise any success, resulting in sentencing under Art. 64 SCC. Persons under indefinite incarceration do not have to undergo psychotherapeutic treatment. However, to have any prospect of release, the person has to receive psychotherapeutic treatment, of which content and progress is also reported to the authorities. The authorities can modify the indefinite incarceration to a measure under Art. 59–61 based on these evaluations. Therefore, if a person sentenced under Art. 64 receives mental health care, MHPs have to report to the authorities if the content is of importance to the authorities decision-making process. In our sample, we included persons sentenced under Art. 64 only if they received mental health care.

Concerning the therapeutic settings, in-patient treatment of a measure should ideally be carried out in a psychiatric or therapeutic institution. However, the person can also be incarcerated in a penal institution given that therapeutic treatment can be provided by specialist staff (e.g., forensic psychotherapists and psychiatrists). The treatment provided will depend on the placement of the person (including the orientation of the institution and the MHP) but also on the type of offense committed and mental health condition. It is therefore not possible to characterize the types of therapies, that our participants received, in detail. However, it can be said, that in practice, most persons sentenced to measures will at a minimum receive individual psychotherapy sessions at a regular interval (e.g., weekly, biweekly, or monthly). Others additionally receive group therapy and some treatment units might foster a therapeutic encounter throughout the day. The type of institution will not give a reliable account of the treatment provided, as for instance, intense therapeutic treatment units are also available in some penal institutions (see Brägger, 2014 for an overview on placement options for persons sentenced under a measure).

### Data Analysis

The software program MAXQDA was used to support and manage data analysis processes. To build a uniform coding tree for the entire project, eight interviews were first read and coded together by five project members. This allowed the study team to discuss different nuances that are visible in the data and to agree on how to name different codes, and what the codes mean in case of complex code names. Thereafter, three study team members (FP, TW, and HM) individually coded all the remaining transcripts and came together to discuss the new codes, solve disagreements, and sorted the final coding tree. During the entire process, the analysis followed thematic analysis (Braun and Clarke, 2006).

In light of the richness of the data and the broad scope of the interviews carried out for the project, coded data related to dual loyalty and the elicitation technique were extracted and examined in-depth for this article. That is, HM carefully read this sorted data segments in its entirety, re-examined the codes applied to this data extract, and further analyzed them with the study purpose as the focal point. This in-depth analysis on one topic also followed thematic analysis and two major themes were evident “*The perception of the dual loyalty conflict*” and “*Developing a trustful relationship to address dual loyalty conflicts*.” Examples of coded quotations were chosen by HM and TW to illustrate the below presented themes. HM translated the codes from the original language into English, the translations were checked by an English native speaker. All authors agreed to the results presented in this article and its interpretation.

## Results

**FIGURE 1:**
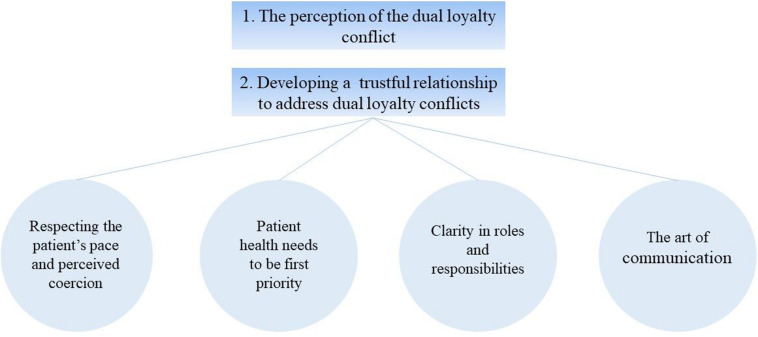
Main topics on the patients’ perception on their psychotherapist’s dual loyalty conflict.

See Figure 1 for an overview of the below presented topics.

### The Perception of the Dual Loyalty Conflict

All study participants positioned their psychotherapist on the triangle indicating that they were aware of the mental health professionals’ dual role in this setting. Their experiences with psychotherapeutic treatments were very diverse with responses ranging from being highly dissatisfied resulting in treatment discontinuation to highly satisfied and thereby the wish for more therapy sessions.

“What I would like most is not just to go to therapy once a week, but preferably twice a week. Or longer, the session”. (D414)

“I consider him very close to me. Anyway, [Name of psychiatrist] that I’ve had so far, he’s/he’s very good, yes.” (F446)

“And I have often discussed this with the woman [name of the therapist]. Where it was about that I would break off my therapy and so on. That I said: How/I can’t understand that she can stand behind the system. Then afterwards she says that this is her job and to bring the people here so far and that simply at the expense of things that shouldn’t be.” (D429)

**FIGURE 2:**
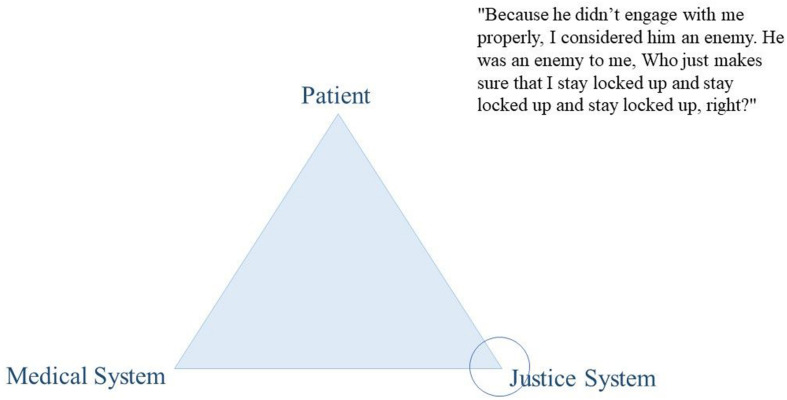
Citation of participant D438.

**FIGURE 3:**
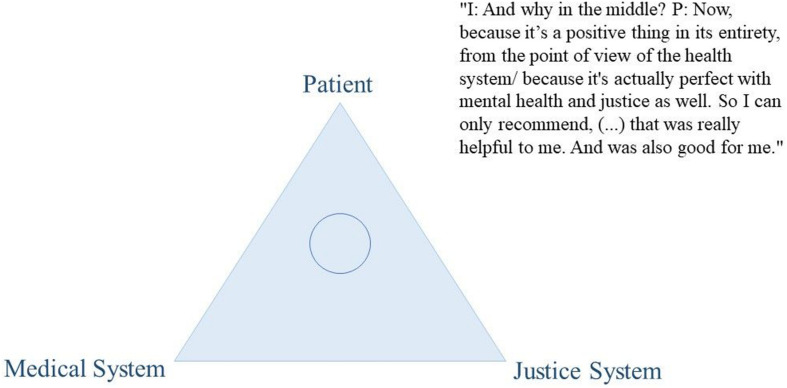
Citation of participant D439.

**FIGURE 4:**
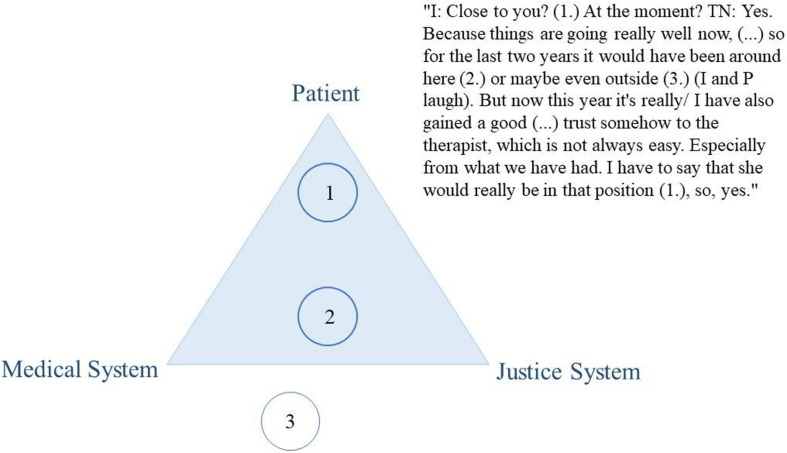
Citation of participant D405.

**FIGURE 5:**
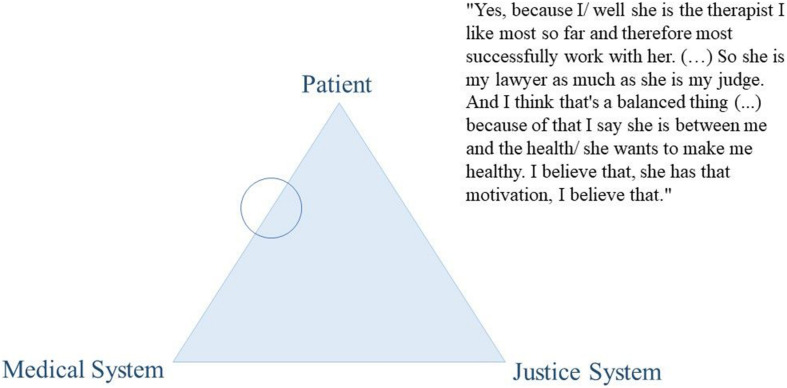
Citation of participant D403.

From our data, this variability in treatment satisfaction was mirrored in participants’ positioning of their therapists on the “triangle graphic” and reflected their overall evaluation of the intervention. Participants’ negative experiences were accompanied by therapists’ positioning close to the justice system (see as an example Figure 2). Conversely, their positive experiences were linked to therapists being positioned on the patient side, the medical side, and in the middle of the triangle (see as an example Figures 3–5).

It is relevant for our analysis to point out that the respondents’ positioning of their treating mental health professional in the triangle did not systematically deviate between the participants from both language regions or between types of institutions. Treatment satisfaction and the perception of the dual loyalty conflict did not depend on the language region, in which the participants were imprisoned. Responses neither differed based on the participant’s placement in a psychiatric or penal institution.

### Developing a Trustful Relationship to Address the Dual Loyalty Conflict

Participants were asked to describe their reasons for the positioning of their therapist and for characterizing the therapy as an overall beneficial or rather as an adverse experience. The reasons provided were based on whether the participants felt that they could trust the therapist or whether they felt betrayed by the therapist. For instance, the following participant (who positioned his therapist close to himself) stated that at that moment he was happy with the progress of his therapy (positive overall evaluation) and shortly thereafter emphasizes that he gained trust in his therapist, which he did not consider as an easy process:

“I: Close to you? Right now? P: Yes. Because, it’s going really well now, (…) I’ve gained a good, uh yes, trust too, somehow to the therapist, which is not always easy.” (D405)

The majority of participants who positioned the therapist close to themselves mentioned that it was because they trusted their therapist, as succinctly phrased by participant D438**:** “We have a professional distance, indeed, but it is very close in terms of trust.” A few respondents even described their therapist as the only person within prison walls whom they relied on, describing their therapist as their sole safety net during imprisonment. F409 stated: “She’s the only person here in the facility that I can really open up to because there’s no one else whatsoever.” In contrast, the majority of respondents, who positioned the mental health professional close to the justice system, linked it to a lack of trust:

“He may not be happy [to hear this] but he’s closer to the justice. I: And why do you say he might not be happy? P: No, because it could be a lack of trust but… yes he knows that! (…) Well, I always said he had two hats on, he has the psychiatrist’s hat plus the one of justice! So, (…) since he’s responsible for the prison thing, I’m a little bit suspicious of him anyway.” (F441)

Several other respondents, who positioned their therapist close to the justice system, reasoned that they had the impression the therapist would work against them. For instance, they expressed the feeling that the psychotherapist was searching for reasons to keep them imprisoned and that anything they said might be used against them: “So it actually does worry me that something I say to them will come back and be used against me.” (F412).

“Because he didn’t engage with me properly, I consider him an enemy. He was an enemy to me. Who just makes sure that I stay locked up and stay locked up and stay locked up, right?” (D438)

When we asked participants what made them trust or mistrust their therapist, prompting for more specific explanations, they named certain therapist characteristics and strategies that influenced the development of a trustful relationship. These subtopics, that resulted from inquiring on the perception of their therapist’s dual loyalty conflict, are all related to the development and maintenance of the therapeutic alliance, which we grouped into four themes: (1) respecting the patient’s pace and perceived coercion; (2) patient health needs to be first priority; 3) clarity in roles and responsibilities; and (4) the art of communication – between transparency and unchecked information sharing.

#### Respecting the Patient’s Pace and Perceived Coercion

Several participants noted that they showed resistance at first and that it took a lot of time to gain trust in their treating therapist. Some said it took a few weeks, others indicated it took three to 6 months. Overall, many stated that they needed time to get to know each other, as outlined by the following participant:

“It took about half a year to get there. We had to get to know each other first and uh, yes, have certain conversations, observe, or yes. And also, I had to see, if I/if I address something now, how does she react? How does she behave?” (D438)

Several respondents further elaborated that it was central to have a sense of control over the situation and that they themselves were the pacemakers of therapy progression. One participants described this as the therapist trying to approach him/her – and not the other way around:

“I was stubborn, so at first. But, she showed a lot of understanding and told me again and again: ‘Well, I pave the way,’ or and I can talk about what I want to talk about. And yes, that/that casual, but nevertheless groundbreaking, she has found the way to me. So she to ME, not me to her.” (D428)

A few respondents further depicted trust building as a very slow progression, within which they started to realize that the therapy sessions helped them to feel better. They highlighted that even though they were mandated to therapy sessions, it was key, that the therapist did not pressure them but was merely seeking the conversation:

“Yeah, you know, you’re not really forced to lie down on a ‘saltire’ [couch], what am I supposed to lie down on a saltire? [Here] they are just looking for the conversation, so first of all you have to build up trust with the psychologist, or, with the person opposite. Just like I said before, openness has developed more and more, to be able to talk about everything.” (D404)

“Because I myself also noticed that it does me good and in a certain way it does me good and [so I told myself] ‘so now get involved with the new therapist, you have no choice anyway, so try to find a way.”’ (D428)

#### Patient Health Needs to Be First Priority

Several respondents highlighted that they trusted their mental health professional because they developed the impression that the therapist’s goal was to enhance their well-being and to help them to get better. They thus felt that the therapists were not focusing on their role to control and monitor the participants, which was appreciated.

“P: It was more the direction, we are there for you. I: Okay. So the way you position yourself? P: Right. And also say: ‘Okay, I’m here to help,’ independent now/of course the offence plays a role, of course it’s first of all about that, but ‘We look that it goes forward with you, that you go into a direction. That we can help.”’ (F408)

“They see their role as monitoring me, rather than necessarily trying to assist me.” (F412)

This difference in whether the therapist appeared to value control or care for the patient seemed to be linked to feelings of being supported in their individual needs versus following highly structured treatment plans, ignoring the patient’s individuality. More specifically, several respondents who placed their therapist close to themselves stated that the therapist would target their individual needs, fully understand them and their problems, as well as respect them as the person they are. This, they said, enabled them to open up and to share their deepest secrets – to develop a trusting relationship. For instance, two participants depicted that they confided in their treating mental health professional because he/she showed engagement to work on topics that were relevant to the participants: “Precisely because I have faith in her. I can talk about anything, really talk about anything.” (D404), or “She addresses the topics in the right way, or what concerns you and they will be dealt with afterwards.” (D402). One respondent pointed out that he/she trusted the therapist because he/she could be oneself: “Because we trust each other blindly, because we know each other, and because from my side, I can be myself.” (D414). Another respondent who positioned his therapist close to him-/herself highlighted that he/she was able to rely on their psychotherapist to support them in any situation: “With all the lows and highs, and he kept carrying me out. And that’s my life saver, in plain English. Yes, because without him, as well as without certain other people, I would no longer be here. That’s why I want to give him a high value, or in other words, he is close to me.” (D419).

In contrast to this, respondents, who positioned their therapist closer to the justice system, stated that their treatment was highly structured and not respecting their individuality. They perceived the structure and goal of the treatment as predefined by the justice system and described it as impersonal.

“It is much, eh I personally find it more impersonal than it was perhaps ten years ago. It is really more structured and more eh I have to treat this, I have to push that through, I have to do this.” (F408)

#### Clarity in Roles and Responsibilities

A few respondents highlighted that they confided in their mental health professional when their role within the system was devoted to caretaking only and their affiliations with the justice system clearly communicated. For instance, one respondent underlined that it was crucial that all players involved needed to have clear separation of roles and explicit assignment of responsibilities. Having such clarity was meaningful for the patient to understand the course of events and the measures imposed. He criticized that in his case, nobody wanted to take responsibility for anything but referred to others or the system in general:

“He is quite far removed from my interests! He is under the cover of his function, but he is at the service of the legal system. Because every… every decision we make at every level is made in consultation with the legal system, and the legal system prevails among all stakeholders! This is the one that prevails. And those who take… that’s my psychotherapist! But there’s a hierarchy above him. So each one covers himself with one, with the other and there’s no one who takes a real responsibility, a commitment… it’s difficult!” (F445)

Further, participants’ responses indicated that his/her relationship with their therapist was impacted negatively when the mental health professional adopted controlling tasks. For instance, the following respondent describes a situation in which the mental health professionals would specify certain therapy goals and link them with benefits such as temporary or escorted leaves:

“Because the therapists here, they don’t have to explain to me – so this is my personal opinion – they don’t have to explain to me that they are only interested in us. They have a mandate from the justice system and they have to fulfill it. No matter what it costs. It will simply be fulfilled. It’s like a catalogue. These are the expectations tack tack tack tack tack tack tack tack, you have to fulfil them and if you fulfil them, then you can take a step forward and then you can get certain privileges. (…) I can’t understand that the people back the system, that exists here, that the psychologists back such a system, I can’t understand, simply not. (…) then there are those who pass the order from the justice system – because it is easier. Then they do the job handling the privileges, for example, there are many authorities that give it away. So to speak ‘You can deal with the privileges. You can handle the leaves yourself, using our framework.’ They give it away like that. (…) He didn’t care, the one I got, the guy in charge from the authorities, gave the responsibilities of my privileges to here.” (D429)

#### The Art of Communication – Between Transparency and Unchecked Information Sharing

Many participants discussed the importance of well-managed communication. They found communication difficult because as a condition of their mandated psychotherapeutic treatment medical confidentiality is limited. Study participants stated that their therapists would share information with the authorities and other prison and health care staff. They presented three examples, in which transparency was key to build a trustful relationship: (1) breaches to confidentiality, (2) therapist’s authenticity and direct feedback, and (3) protecting patient’s private details.

First, participants who said that they trusted their treating therapist emphasized that transparency about breaches to confidentiality was key. They stated that they appreciated either knowing under what circumstances their information was shared or being asked to consent to the passing of information before the particular situation occurs. This was highlighted by the following respondents:

“[My therapist said] ‘We’re still bound to secrecy, we’re still bound to medical confidentiality, but if by chance we see that you’re not well, or, that you’re telling us something about children or like that, that you have fantasies about children or anything’ – well, they told me ’about that we’re obliged to notify the authorities [Name of Institution] and then…/but that’s fine.” (F450)

“If he passes more information to / the authorities, he always asks my opinion, ‘Can I talk to the authorities about it?’, he always asks me.” (F453)

“Yeah, that’s not what I expected from a psychologist, to go telling these things.” (D404)

This was particularly highlighted in relation to the annual report to the justice system. This written report was named as one of the most common breaches to confidentiality, in which the treating psychotherapist would summarize significant information of the therapy content and progress. The report influences the authority’s decision making on the prolongation of the mandated treatment and further security imperatives. It has therefore a high impact on the patient’s future. Most respondents stated that, even though they could not influence the content of the therapy report, they appreciated getting to read it before it was sent out. They further claimed that it would give them an opportunity to discuss discrepancies with their therapist before it was too late. This gave them a certain degree of control over their own situation, as highlighted by a participant:

“We also read the report first before it goes to the authorities. Of course we have no influence on it but at least we know what goes to the authorities. It’s more transparency, it’s more openness.” (D403)

Second, many respondents found it crucial that the therapist is transparent, i.e., that he/she was open and honest. They stated that the therapist’s feedback to the patient needed to reflect what they believed about the patient’s mental health and their progress in therapy. If this was the case and the therapist’s behavior during treatment was in accordance to the report written to the authorities – this was perceived as very positive and the dual loyalty conflict did not appear to have a negative impact on the treatment:

“She is one who says her opinion, often it is in some therapy reports or something, uh it is so that the therapist has an opinion and then the boss writes his opinion in there and then it is changed and she does not allow that for example.” (D403)

“P: She does not write in my favour but she writes it truthfully, or, and… if she would write it for the justice system, then she would weight it a bit more like, yes, uh, ‘He is so and so far’, but (emphasizes)… but the ‘but’ that is missing, that is nowhere in it, that is simply – honesty is in it. Nothing else. Yes. I: So it matches with what she tells you directly in therapy P: Exactly, that’s also in the report…. – she gives me this to read, if I agree and so on, and then she sends it off. Yes. Yes. I: That means you have the opportunity to talk about it again? P: Exactly, yes. If you would object to something, that you could still discuss it. But now with the last report, I have to say again – it’s one-to-one.” (D402)

Respondents further highlighted that feedback needed to be direct and uttered promptly without much time-delay. If participants felt they were not informed in time or even deceived, this would impact their relation to their therapist and they would feel powerless and at the mercy of the system.

“Afterwards they write a report, then at some point you get the report to read and then it says according to the situation, about me it said, ‘she did not participate’ and there was not so much to write, because I just did not want to. And afterwards/you are at their mercy. And they don’t have to tell me that they care how you are. They have a mandate from justice and they have to do it.” (D429)

“She just – it/the stuff that she/it has picked up about me, has passed it to other psychosocial support staff and stuff and stories they have twisted that seven times and so on and, yeah…. That’s what takes your trust afterwards, right. (…) And after that I just said: ‘If they see something that I have done something that is not fair to the others, then come and tell me’, you can’t punish a dog two weeks later, he wouldn’t remember it anymore either. And not just say ‘Yes, the team saw it.”’ (D402)

Third, participants did not appreciate when they had the impression that their information was shared with either people that were not directly involved in their health care or in non-structured settings. For instance, one participant highlighted that security personnel was informed about private details with the excuse to create more security.

“The whole thing is much more transparent under the pretext of security of course, they always say: ‘As soon as we know what medication he takes, what problems he has, the more we can react as security officers.’, they would then say. In the sense that afterwards of course a lot, a lot, a lot is taken under the hat … secrecy, that the cease/uh in principle no longer exists.” (F408)

Other participants pointed out, with disappointment, that personal information was at times shared at lunch with other staff or in the group room where prisoners and therapists meet all together. Consequently, far more people who were not concerned with their case heard about their details, as indicated by respondent D429: “…if they sometimes discussed things like that over lunch.”

## Discussion

This interview study is important in that it obtained qualitative data from incarcerated persons receiving court-ordered therapy, in particular concerning patients’ perception of the therapist’s dual role in the case of court-mandated treatment. First, our participants’ perception of the clinician’s involvement with the justice system was linked to their overall treatment experience. Their affiliation with the justice system was mentioned as important factor that affects patient’s treatment satisfaction. It is therefore crucial how mental health professionals describe and deal with the influences of the justice system when providing therapy to patients. Second, study findings indicate that for the respondents it is central that therapists take up an exclusively caretaking role and pursue the objective of enhancing the patient’s health. To achieve such clarity in roles and responsibilities, mental health professionals must communicate their affiliations with the justice system transparently. This requires therapists to explain upfront and to disclose breaches to confidentiality, to display authentic behavior, and to provide direct feedback to the patient. It is further important for clients that therapists respect patients’ individuality and personal needs, and advance in line with the pace of the patient. Participants perceived this as key, not only to build a trustful relationship but also to be motivated to engage in treatment. Our results therefore support previous claims, that “even if the framework of a relationship can be imposed, the trust cannot be forced” (Lagarde and Msellati, 2017, p. 5).

Our findings indicate that for patients it is crucial that their individual needs are acknowledged and respected. This is in line with earlier research underlining the importance of therapists’ flexibility in responding to client individuality to develop a therapeutic alliance and influence treatment outcome (Marshall and Serran, 2004; Gannon and Ward, 2014; Blasko et al., 2018; Jeglic and Katsman, 2018). However, it stands in contradiction to the use of highly structured manuals that suppress flexibility and neglect client individuality but are common for treatments following the risk-need-responsivity model developed by Andrews and Bonta (2010). Our participants’ responses therefore support the recent shift to a greater focus on client individuality that are inherent to strength-based approaches such as the Good Lives Model (see for example, Ward and Gannon, 2006). Nevertheless, this does not necessarily stand in contradiction to the risk-need-responsivity model as the responsivity principle emphasizes the importance that the treatment needs to fit the client’s ability and learning style. It requires the therapist to adapt a flexible style in order to recognize and respond to topics and goals important to the client (Marshall and Serran, 2004). However, even though our participants’ associated highly structured programs with a lack of individuality, it might just show how difficult it can be to balance manual rigidity with patient individuality – and this challenge remains with the psychotherapist.

Our results inform that the therapist’s ability to recognize and respond to patient’s needs is particularly important during early psychotherapy sessions. Study participants stated that, to overcome initial resistance, it was important to have a sense of control over the content and pace of therapy, to gain trust in their treating therapist. Earlier research has highlighted that in offender therapy it is crucial to take one’s time to overcome mistrust to establish an effective alliance (Marshall and Serran, 2004; Goulet et al., 2019). This might be a particularly important aspect in therapy with persons in detention, as research in the community has shown that patients build up comparable alliance levels after three to five sessions on average (Zimmermann et al., 2019), in contrast, our participants’ indicated taking three to 6 months time. It might be particularly difficult to build a strong alliance with patients suffering from substance use or personality disorders (Ross et al., 2008; Meyer et al., 2019), two highly prevalent disorders within the correctional context (Fazel et al., 2016). However, it still needs to be clarified whether and how the development of a strong alliance might differ between community patients and persons living in detention. Other therapeutic alliance research concludes that therapists and clients need to align their treatment expectations and goals to develop a collaborative working alliance (Fluckiger et al., 2015). The identification of the patient’s needs and wishes is therefore a cornerstone of a trusting and collaborative relationship.

Our results provide evidence that mental health professionals working in correctional context should emphasize their role as supportive caretakers to establish a high quality alliance. This is particularly important, as mental health professionals who are able to create a warm, caring, and supporting environment have been shown to be more effective at facilitating change (Marshall et al., 2003; Blasko et al., 2018). It is further crucial, as mental health professionals are frequently even described as the only supporting person within prison walls (Skeem Louden et al., 2009). A person’s deprivation of freedom is accompanied by a removal of his/her social network. At the same time, it is widely known that positive and strong bonds with others are central for one’s well-being (Turner and Brown, 2009). The patient’s relationship with the therapist might consequently be of greater meaning to a patient in prison compared to a patient outside the walls. This stresses the importance to facilitate conditions that enable the patient and the mental health professional to form a strong and trusting relationship in correctional contexts.

Further, our study results support previous findings that mental health professionals should not be directly involved in punitive control (Marshall and Serran, 2004; Hachtel et al., 2019; Wittouck and Vander Beken, 2019). To clear doubts related to their association with the justice system, mental health professionals must transparently discuss their role and affiliation with the justice system with their patients. Our study participants appreciated such role clarity and accepted the therapists’ duty in sharing information with the authorities. These findings support the procedural justice principle of “information,” which states that patients need to receive information and clarification about procedures (Wittouck and Vander Beken, 2019). However, our study participants underlined that mental health professionals’ tasks and responsibilities should concern mental health care only, which would create a rehabilitative environment and the grounds for a trusting relationship. Simultaneously, if the patient knew who was responsible for the “controlling” aspects, it facilitated the perception of the mental health professionals as taking up the “caring” role.

The question remains how to ensure that mental health professionals exclusively carry out caretaking roles. In the mandatory treatment setting, by definition the therapist holds the double role to care and control. Our findings suggest that all patients need clear information about the roles and obligations of health professionals working in prison: when the different professions working with patients undergoing mandated therapy have clearly and distinctly assigned roles and are at the same time in close contact with the patient, it is possible to build a trusting relationship in spite of the constraints posed by correctional contexts. Thus, as also stated by Strasburger et al. (1997), if there are different roles and responsibilities, then they should be assigned to different players, making each person wearing its own hat.

All our participants reported dual role conflicts of their mental health professionals. This was irrespective of the language region they belonged to or the specific setting they were housed in. This suggests that the fact that a mental health professional works with a patient, who is mandated to psychotherapeutic treatment, might be a reason enough for a patient to doubt his or her independence. The reason that we did not see any strong differences between settings and cultural embedding might, however, also lie in the nature of our qualitative approach. Quantitative analyses might be able to detect differences between specific setting-related factors more precisely and should be the focus of future research.

Trustworthiness has been established as an important therapist characteristic to promote a high quality alliance (Ackerman and Hilsenroth, 2003; Hilsenroth et al., 2012; Fluckiger et al., 2018). In court-mandated treatment orders, trust is at stake due to limited confidentiality. Gannon and Ward (2014) highlighted that mental health professionals are frequently asked to share treatment information with the authorities depicting a “common correctional challenge to the therapeutic relationship.” Our research supports earlier findings that the conditions of limited confidentiality and its implications for the patient need to be explained transparently (Gannon and Ward, 2014; Elger et al., 2015a, b; Merkt et al., unpublished).

Thus, there is an important need to ensure transparency in the therapy context, emphasized in the subtheme on communication. First, transparency requires information sharing with other staff in structured and confidential settings. Patients’ private details should be shared exclusively with professionals that are directly involved with the patient care. Second, information that is passed to the authorities (in our study in form of a yearly written report), needs to be previously shared with the patient (Canela et al., 2019). Mental health professionals, who share their report prior to sending it to judicial authorities, allow participatory decision-making to take place. This has been indicated to be linked to less perceived coercion (Hachtel et al., 2019) and reduced violations in involuntary settings (Skeem Louden et al., 2009). In addition, it provides empirical evidence for the “voice” principle of the procedural justice theory, as sharing the report allows the patient to express their own view (Wittouck and Vander Beken, 2019).

Moreover, the content of the report needs to be in line with the mental health professional’s on-going feedback during therapy. When the therapist undertakes such measures, the report does not appear surprising to the patient since the therapist was genuine. That is, he or she was authentic and honest throughout treatment and the report is thereby a reflection of the therapy content. Genuineness is well-established as an important therapist characteristic in general psychotherapy (Nienhuis et al., 2018). Also, sharing the report provides an opportunity to estimate the degree of agreement between the patient and the therapist. Gelso (2014) states that the therapeutic relationship is “marked by the extent to which each is genuine with the other and perceives/experiences the other in ways that befit the other”: This “sharing” procedure could therefore be an opportunity to review and increase the strength of the bond between the mental health professional and the client.

### Limitations

Our study followed a qualitative study design, which involves limitations inherent to this methodology. First, our participants’ responses might have been influenced by their desire to utter socially accepted opinions. Participants might think that researchers are linked to the justice system, that anonymity is not provided, or that their participation and responses provided during the interviews might alter their chance for release (Dugosh et al., 2010; McDermott, 2013). These perceptions have the potential to alter their responses toward more socially accepted opinions, thereby creating concerns related to validity and reliability (Copes et al., 2013). For these reasons, we also did not collect systematically demographic characteristics such as index offense, time in prison, and psychiatric diagnosis during the interviews unless they were shared voluntarily.

Second, the participants’ responses might have differed due to their mental health issues. For instance, patients with problematic personality traits might have more difficulties in establishing an alliance with their treating psychotherapist compared to other patients (Ross et al., 2008; Kennealy et al., 2012). The differences in the perception of the dual loyalty conflict could therefore be linked to the psychiatric diagnosis and could be unassociated with therapists’ abilities to deal with the dual loyalty conflict.

Third, older incarcerated participants were recruited through contact persons of the participating correctional institutions thereby raising the issue of potential volunteer as well as selection bias. Therefore, we might have attracted older incarcerated persons with a certain set of opinions and their opinions may vary from younger incarcerated persons.

Fourth, our participants were imprisoned in Swiss correctional institutions. Our results are therefore limited to this specific context and are not generalizable to other contexts. However, we included persons living in detention from two different language regions and different types of correctional institutions and therefore believe that we covered the prevailing notions on the perceived dual role conflict in this context. Most importantly, our findings are based on participants’ own reports identifying a range of experiences with their therapists. These findings were not limited to predefined experiences, as might occur in a survey-based research.

### Future Research

Based on our findings and our overall methodology used, we forward the following to improve our understanding on this topic. First, we interviewed persons living in detention in 14 different institutions from the German and French speaking language regions. Our participants were therefore subject to differing settings and treatment options. This recruiting strategy allowed us to shed light into notions and experiences that are independent of the specific setting. This is particularly important within the Swiss context, as communication between language regions is often hampered. Our research project therefore also aimed at generating knowledge bypassing language barriers and looking at commonalities between the regions. This, however, increased the heterogeneity of our sample, particularly, in regards to types of mental health care received. As for example, some participants received individual sessions only while others were embedded in a more holistic program of a specialized treatment unit. Further research should therefore investigate the impact of certain treatment settings and orientations on the perception of the MHP’s dual role.

Second, we did not gather detailed information on the duration and orientation of treatment and conducted only one interview on a single occasion. However, the perception of the alliance can change throughout therapy. As for instance, the development of an alliance will require time in the beginning of therapy and ruptures during on-going therapy need repairing. Thus, the time point within the participant’s therapy sequences and the fact that we conducted one interview only might have affected the responses. Future research should therefore explore the link between the perception of the dual role conflict and the development and quality of the alliance over time.

Third, we did not collect data on the MHPs’ expertise and qualification. A MHP’s experience, training and skills has an impact on their ability to build and repair therapeutic relationships (Roos and Werbart, 2013). As we allowed the participants to elaborate on current treatment experiences as well as to draw comparisons with previous treatment experiences, information on the current treating MHPs expertise would have not added any value to our data. Future research should consider assessing the clients’ perceptions linking them with demographic and professional characteristics of the MHPs.

## Conclusion

Developing a high quality alliance in mandatory treatment with persons in detention is central due to its relationship with recovery and desistance. Our findings show that some therapists’ characteristics and activities attenuate the negative impact of their double role on the development and maintenance of the alliance. Patients valued a well-managed care-control balance that was characterized by (a) prioritizing patients well-being over security aspects, (b) providing transparency in regards to conditions of the court-mandated treatment setting (e.g., limits to confidentiality, MHP’s interaction with representatives of the justice system), (c) ascribing the controlling role to a separate person who is tangible (e.g., responsibilities are clearly distributed and every involved person is accessible and known to the patient), and (d) showing some flexibility to take the patient’s individuality into account. To increase the effectiveness of court-mandated treatments, we need to support clinicians in dealing with their dual role to allow the formation of a high quality therapeutic alliance. Our qualitative interview study contributed to this much-needed empirical research on therapist’ characteristics promoting a trusting relationship in correctional settings.

## Data Availability Statement

The raw data supporting the conclusions of this article will be made available by the authors, without undue reservation.

## Ethics Statement

The studies involving human participants were reviewed and approved by the Ethikkommission Nordwest- und Zentralschweiz. The patients/participants provided their written informed consent to participate in this study.

## Author Contributions

HM contributed to the data collection and analysis, drafted the first version of the manuscript, and improved the different draft based on the co-authors comments and suggestions. TW made substantial contributions to the study conception, data analysis, and revision of the work. FP, ML, and CD provided substantial contributions by examining the results presented in draft versions and revised the work. BE provided substantial contributions to the study conception and revision of the work. All authors agreed to the final draft of the manuscript submitted and take responsibility for its content.

## Conflict of Interest

The authors declare that the research was conducted in the absence of any commercial or financial relationships that could be construed as a potential conflict of interest.
